# Effects of p53-knockout in vascular smooth muscle cells on atherosclerosis in mice

**DOI:** 10.1371/journal.pone.0175061

**Published:** 2017-03-31

**Authors:** Richard Yang Cao, Robert Eves, Lilly Jia, Colin D. Funk, Zongchao Jia, Alan S. Mak

**Affiliations:** Department of Biomedical and Molecular Sciences, Queen’s University, Kingston, Ontario, Canada; Universitatsklinikum Freiburg, GERMANY

## Abstract

*In vitro* and *in vivo* evidence has indicated that the tumor suppressor, p53, may play a significant role in the regulation of atherosclerotic plaque formation. *In vivo* studies using global knockout mice models, however, have generated inconclusive results that do not address the roles of p53 in various cell types involved in atherosclerosis. In this study, we have specifically ablated p53 in vascular smooth muscle cells (VSMC) in the ApoE^-/-^ mouse model to investigate the roles of p53 in VSMC in atherosclerotic plaque formation and stability. We found that p53 deficiency in VSMC alone did not affect the overall size of atherosclerotic lesions. However, there was a significant increase in the number of p53^-/-^ VSMC in the fibrous caps of atherosclerotic plaques in the early stages of plaque development. Loss of p53 results in migration of VSMC at a faster rate using wound healing assays and augments PDGF-induced formation of circular dorsal ruffles (CDR), known to be involved in cell migration and internalization of surface receptors. Furthermore, aortic VSMC from ApoE^-/-^ /p53^-/-^ mice produce significantly more podosomes and are more invasive. We conclude that p53^-/-^ VSMC are enriched in the fibrous caps of lesions at early stages of plaque formation, which is caused in part by an increase in VSMC migration and invasion as shown by p53^-/-^ VSMC in culture having significantly higher rates of migration and producing more CDRs and invasive podosomes.

## Introduction

Differentiated VSMC comprise an array of contractile myosin-actin filaments for maintenance of vascular tone. In response to endothelial injury, circulating inflammatory cells such as macrophages and T-lymphocytes are recruited and trigger a switch of VSMC phenotype from contractile to synthetic/proliferative with migratory and invasive potential [1,2]. Media-to-intima migration of VSMC in the arterial wall is a hallmark characteristic of atherosclerosis and plaque formation. This is achieved by remodelling of the actin cytoskeleton to produce actin-based membrane protrusions such as ruffles and podosomes [3–7]. Release of proteases by podosomes, primarily matrix metalloproteases (MMPs), enables digestion of extracellular matrix (ECM) proteins, hence clearing a path for migration [8]. Thus, VSMC migration and invasion of ECM are highly coordinated processes, but not necessarily under the same regulatory mechanisms.

The p53 transcription factor is well documented for its roles as a potent tumor suppressor that regulates cell cycle progression and apoptosis [9]. We have shown *in vitro* that p53 also acts as a suppressor of cell migration and invasion in VSMC by down regulating the formation of circular dorsal ruffles (CDR) and podosomes [3,5,6,10,11]. A number of studies have been attempted to explore the anti-proliferative and pro-apoptotic roles of p53 in atherosclerosis using genetically engineered mouse models [12–20]. The first *in vivo* study was reported 15 years ago by Guevara et al [12] who showed that ApoE^-/-^/p53^-/-^ double knockout mice fed a Western diet had up to 2-fold increase in aortic lesion area that was attributed to an increase in cell proliferation rather than a decrease in apoptotic cells in lesions.

Global p53 knockout approaches in animal models of atherosclerosis have provided important and sometimes unexpected information about cell proliferation and apoptosis, especially of macrophages, but have not addressed the role of p53 in specific cell types in plaque formation. Furthermore, none of the *in vivo* studies have addressed the role of cell migration and invasion in atherosclerosis. For these reasons, our goal in this study was to specifically study the role of p53 in cell migration and invasion of VSMC in the biogenesis of atherosclerotic lesions. To this end, we have used Cre-loxP methodology to generate mouse strains in the ApoE^-/-^ background for tamoxifen-inducible ablation of p53 in VSMC using a smooth muscle α-actin promoter.

## Materials and methods

### Generation of mouse strains

**Fig 1:**
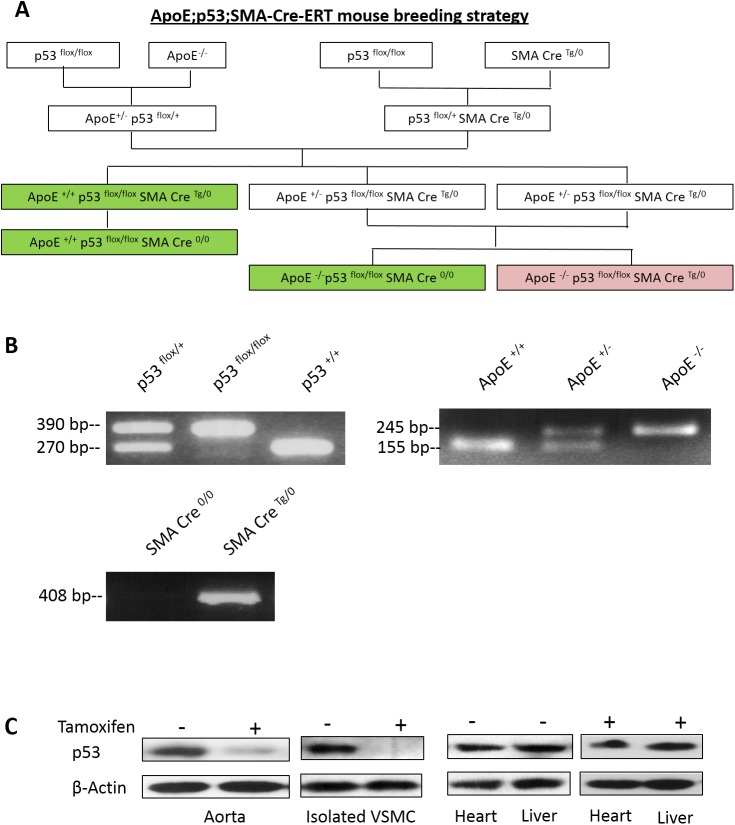
Generation of double knockout of ApoE and smooth muscle specific p53 mice on a C57BL6 background (A) Schematic of the breeding protocol to generate the desired genotypes used in this study. White boxes indicate intermediate genotypes, green boxes indicate control genotypes and pink box indicates double knockout mice. (B) PCR genotyping for three alleles. (C) Whole aorta, liver, heart and cultured smooth muscle cells from ApoE^-/-^ /p53^-/-^ (tamoxifen-treated) and ApoE^-/-^ /p53^flox/flox^ control mice (without tamoxifen) were lysed and analyzed by Western blot to confirm targeted p53 knockout in smooth muscles cells.

All animal procedures were carried out in accordance with CCAC guidelines and Queen’s University Animal Care Committee (UACC), Kingston, Ontario, Canada specifically approved this study (protocol: Mak-2011-002-Or-A1).In order to ameliorate suffering, the health status of mice was monitored daily by animal care staff and if mice were found to be experiencing undue suffering, they were euthanized immediately. Mice on a C57BL/6 genetic background p53 Flox (stock 8462 Jackson Laboratories), Apoe^tm1Unc^ (stock 2052 Jackson Laboratories) or αSMA-Cre-ER^T2^ (Pierre Chambon, IGBMC Strasbourg, France) were crossbred to create the various genotypes required for this study (Fig 1A). Mice (ApoE, p53 and Cre recombinase) were genotyped by PCR analyses using primers and protocols from The Jackson Laboratory. Mice were kept on a 12-hour light/dark schedule for the duration of the study and fed normal mouse chow (5015, PMI, St. Louis Missouri). Six week-old mice were intraperitoneally injected with five daily injections of 1 mg tamoxifen to induce p53 ablation as previously described [16,21]. At the age of 7 weeks, experimental mice were switched to a Western diet (TD88137, Harlan Teklad) for 6, 10 or 15 weeks as indicated. Food and water were available *ad libitum*.

### Antibodies and reagents

The following antibodies were used in this study: Cy3-conjugated smooth muscle α-actin, β-Actin, (Sigma-Aldrich); p53, PDGFRα (Cell Signaling); and cortactin (Fisher Scientific). PDGFβ/β, phorbol-12-13-dibutyrate (PDBu), Oil Red O lipid stain, tamoxifen, and paraformaldehyde were from Sigma-Aldrich. Dissection plates were made from Sylgard 184 (Dow Corning), and Norit SA was from Fisher Scientific.

### Mouse dissections and en face imaging of Oil Red O-stained aortas

Mice, euthanized with CO_2_, were dissected and perfused with PBS. The aorta was removed from the heart to the iliac bifurcation as along with 2 mm of the left common carotid artery and left subclavian artery, and fixed in 1.6% paraformaldehyde. The aorta was cleaned, dissected en face and stained in 0.5% Oil Red O solution for 5 minutes, de-stained in 70% ethanol washes and pinned on a Sylgard plate for imaging on a Leica MZ16 microscope with an Olympus Q-Color 5 camera and Image Pro Plus software.

### Immunostaining of frozen sections and whole aortas

PBS-perfused brachiocephalic arteries were removed from mice and placed in OCT media (Fisher Scientific) and frozen at -80°C. Sections (6–8 μm) were prepared using a Leica CM1900 cryostat and placed on superfrost slides (Fisher Scientific). Slides containing tissue sections, primary cells or whole sections of aorta were washed in PBS, fixed for 5 minutes in 2% paraformaldehyde, permeabilized in Triton X-100 for 5 min, washed 4 times in PBS, stained for 2 hours with Cy3-conjugated smooth muscle α-actin in 3% BSA-PBS, washed 4 times with PBS and mounted under a 12 mm circular glass coverslip with fluorescent mounting media (Dako) containing DAPI (Invitrogen). The ratio of smooth muscle cells to lesion size was determined by measuring the area of each using Image Pro Plus 6 software (Media Cybernetics, Rockville, MD, USA).

### Fluorescence microscopy

Images were taken with a Zeiss Axiovert S100 fluorescence microscope (Toronto, ON, Canada) equipped with a Cooke SensiCam CCD camera (Optikon, Guelph, ON, Canada) with a Plan-Neofluar 40x objective operated by Slidebook 4.3 software (Intelligent Imaging Innovations, Denver, CO, USA). Whole aorta sections were imaged using Quorum Wave Effects Spinning Disc Confocal (Quorum Tech. Guelph, Ontario) controlled by Metamorph software (Molecular Devices, Sunnyvale California).

### Primary aortic smooth muscle cell culture

Aortic smooth muscle cells were isolated from different experimental mouse strains as previously described [10,22]. Cells were then cultured in high-glucose Dulbecco's modified Eagle's medium (DMEM) (Invitrogen), supplemented with 10% bovine growth serum (BGS) (HyClone). Cells were grown in an incubator at 37°C in the presence of 5% CO_2_. Cells were maintained at sub confluency (approximately 80–90%). Cells were assayed by Western blot and immunostained for SMα-actin to determine cell line purity and p53 to ensure correct genotype. Cells were cultured on 12 mm glass coverslips overnight and treated with 2 μM PDBu for 30 or 60 min or serum- starved overnight and treated with 20 ng/ml PDGF for 10 min before staining as previously described [23]. For time-lapse imaging, cells were cultured on ΔT dishes (Bioptechs) and serum starved overnight. Media was replaced with DMEM containing HEPES buffer and no Phenol Red for time lapse imaging every 30 seconds for 60 min.

## Results

### Generation and genotyping of SMC-targeted p53-knockout mice in the ApoE^-/-^ background

The breeding strategy used in the generation of SMC-targeted ApoE^-/-^/p53^-/-^ mice is shown in Fig 1A. The ApoE^-/-^/p53^flox/flox^ /SMA Cre^Tg/0^ mice with or without tamoxifen-treatment were used for subsequent studies along with control mice: ApoE^+/+^/p53^flox/flox^/SMA Cre^Tg/0^, ApoE^+/+^/p53^flox/flox^ /SMA Cre^0/0^, with and without Tamoxifen-treatment, to serve as control for effects of p53-knockout alone on lesion formation. ApoE ^-/-^ /p53 ^flox/flox^ /SMA Cre^0/0^, with or without Tamoxifen-treatment, were used as controls for the effects of Cre and Tamoxifen independent of p53 knockout. We have not detected phenotypic difference among different control mice when compared to ApoE^-/-^/p53^flox/flox^ /SMA Cre^Tg/0^ without Tamoxifen, indicating that genetic manipulations did not affect the phenotype of various control mice used in this study.

Mice were genotyped using PCR as shown in Fig 1B. Homozygous alleles of p53 and ApoE are represented by single bands for p53^flox/flox^ (390 bp), p53^+/+^ (270 bp), ApoE^+/+^ (155 bp) and ApoE^-/-^ (245 bp), while heterozygous alleles are indicated by the presence of both bands for each allele. Only heterozygous alleles were generated for SMA Cre^Tg/0^ as indicated by a single band at 408 bp.

Half of the mice cohort at the age of six weeks were treated with tamoxifen for 5 days to induce SMC-specific knockout of p53 (ApoE^-/-^/p53^-/-^), and the other half were used as controls without tamoxifen treatment (ApoE^-/-^/p53^flox/flox^). Western blots in Fig 1C show that p53 expression is reduced by about 90% in lysates from whole aortas of tamoxifen-treated mice compared to those in control mice without treatment. Since p53 is absent in isolated aortic smooth muscle cells, it shows that p53 ablation was successfully targeted in VSMC that express the SM α-actin promoter. This is further confirmed by the observation that p53 expression is unaffected by tamoxifen treatment in the heart and liver.

### Smooth muscle-specific knockout of p53 does not affect the overall size of atherosclerotic lesions

At the age of 7 weeks, ApoE^-/-^/p53^-/-^ and ApoE^-/-^/p53^flox/flox^ mice were fed a Western diet for up to 15 weeks to enhance atherosclerotic lesion formation. No overt difference in phenotype was observed for the ApoE^-/-^/p53^-/-^ and ApoE^-/-^/p53^flox/flox^ mice (data not shown). Some ApoE^-/-^ mice, however, did develop skin sores that is a known occasional phenotype for these mice [24].

**Fig 2:**
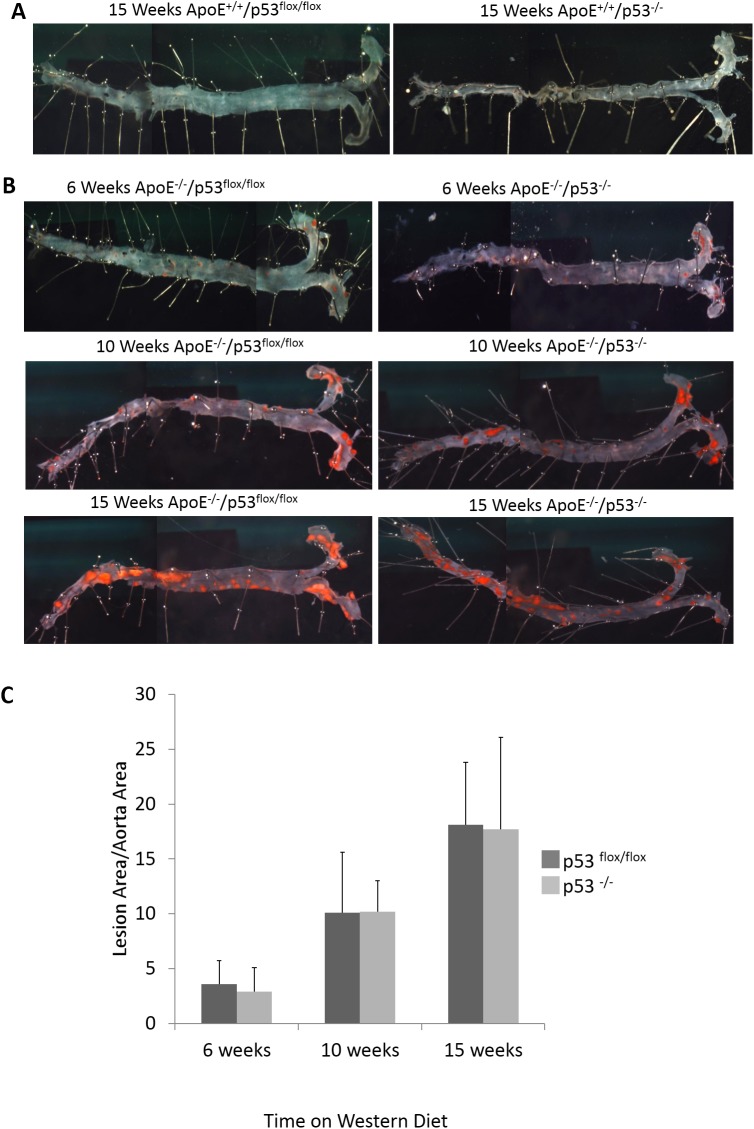
Smooth muscle-specific knockout of p53 does not affect the overall size of atherosclerotic lesions Images of en face aortas stained with Oil Red O lipid stain to show atherosclerotic lesions. (A, B) Aortas from the aortic root to the iliac bifurcation were excised from p53^-/-^ and control p53^flox/flox^ mice in ApoE^+/+^ (A) or ApoE^-/-^ (B) backgrounds. (C) shows the ratio of lesion area (Oil Red O stain) to total aorta area. Error bars represent standard deviation. * indicates p value <0.05, n = 10 per condition.

Next, we investigated the effect of p53-ablation in VSMC on the gross structure of atherosclerotic lesions in aortas of mice fed a Western diet for up to 15 weeks. As shown in Fig 2A, ApoE^+/+^ mice did not develop aortic lesions as indicated by lack of Oil Red O lipid-staining, even after 15 weeks on the Western diet regardless of the p53-expression status. This shows that p53-knockout in smooth muscle cells alone is not sufficient to promote plaque formation in these mice. In ApoE^-/-^ mice, however, atherosclerotic lesions are visible at 6 weeks after the beginning of the Western-Style diet, and increase in size over the 10- and 15-week periods (Fig 2B and 2C) in both p53^-/-^ and p53^flox/flox^ mice. The loss of p53 in VSMC had no significant effect on the formation and overall size of the lesions based on Oil Red O staining (Fig 2B and 2C).

### p53-ablation increases smooth muscle cell population in the fibrous caps of aortic atherosclerotic plaques

We next examined the distribution of SMC in atherosclerotic plaques of p53^-/-^ and p53^flox/flox^ mice, especially in the fibrous cap regions that play a major role in plaque stability [25].

**Fig 3:**
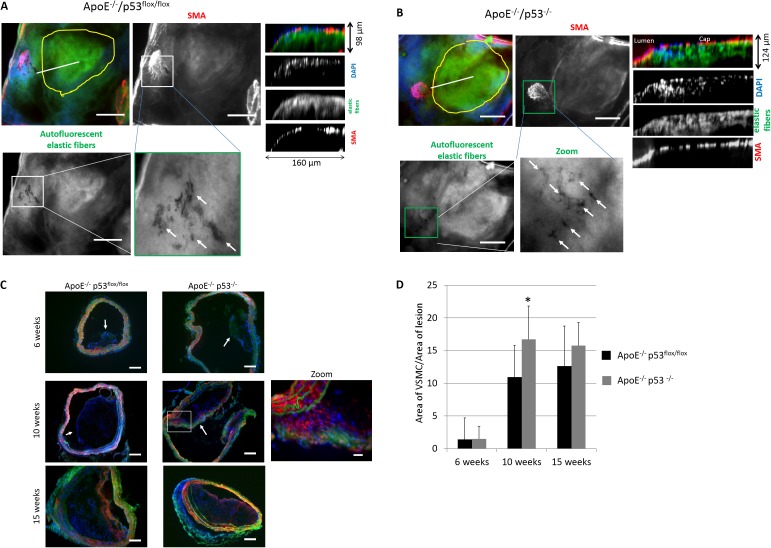
p53 suppresses VSMC-directed fibrous cap formation (A-B) Sections of the aortic arch containing an entire lesion were excised, cut en face and immuno-fluorescently stained for Cy3-conjugated SMα-actin (red), DAPI for nucleus (blue) and elastic fibers, which autofluoresce green. The yellow outline indicates the borders of the lesion, the white line indicates the region of cross-section for images in the third column, white arrows point to areas of degradation of the elastic fibers and the green box outlines the area for the zoom. Scale bars represent 100 μm. (C) Frozen sections of the brachiocephalic artery were immuno-fluorescently stained for Cy3-conjugated SMα-actin (red), DAPI (blue) and elastic fibers (green). The white box indicates the area where the zoom image was taken. Scale bars represent 100 μm or 20 μm for zoom image. Arrows indicate position of atherosclerotic plaques. (D) VSMC cell content was determined from fluorescent stained frozen sections by taking the ratio of the area of the VSMC compared to the total area of the lesion. Error bars represent standard deviation. * indicates p values <0.05.

In Fig 3A and 3B, an area in the aortic arch containing lesions from the ApoE^-/-^/p53^flox/flox^ and ApoE^-/-^/p53^-/-^ mice, respectively, show a layer of VSMCs in the fibrous caps of the plaques. Interestingly, the fibrous caps in ApoE^-/-^/p53^flox/flox^ mice (Fig 3A, 3C and 3D) contain significantly less SMCs than their ApoE^-/-^/p53^-/-^ counterparts (Fig 3B). Clusters of holes can be seen occasionally in the elastin layer under the VSMC adjacent to the lesion (Insets to Fig 3A and 3B). These holes resemble areas of digested ECM seen in *in vitro* assays of podosome-induced ECM degradation, and may represent an imprint of VSMC invasion of the internal elastic lamina and basement membrane *in vivo* during early lesion formation.

To examine the time of appearance of VSMC in the aortic lesions, cross-sections of the brachiocephalic artery of mice, an area of early lesion development [26], was imaged 6, 10 and 15 weeks after Western diet feeding. As shown in Fig 3C, small lesions containing very few VSMC began to appear in the brachiocephalic arteries in some of the ApoE^-/-^/p53^flox/flox^ and ApoE^-/-^/p53^-/-^ mice after 6 weeks. At 10 weeks, VSMC began to appear in the fibrous caps of lesions of the p53 knock-out mice (ApoE^-/-^/p53^-/-^), but significantly less so in the ApoE^-/-^/p53^flox/flox^ animals (Fig 3C and 3D). The number of VSMC in the fibrous caps and plaques plateaued at 15 weeks for the ApoE^-/-^/p53^-/-^ mice, but continued to rise for the ApoE^-/-^/p53^flox/flox^ mice (Fig 3C row 3 and 3D). This finding suggests that the p53-null VSMC respond early to atherosclerotic stimuli and migrate from the medial to intimal layer contributing to early lesion formation and subsequently to position at the fibrous caps.

### p53-knockout in VSMC augments PDGF-induced cell migration and circular dorsal ruffle formation

**Fig 4:**
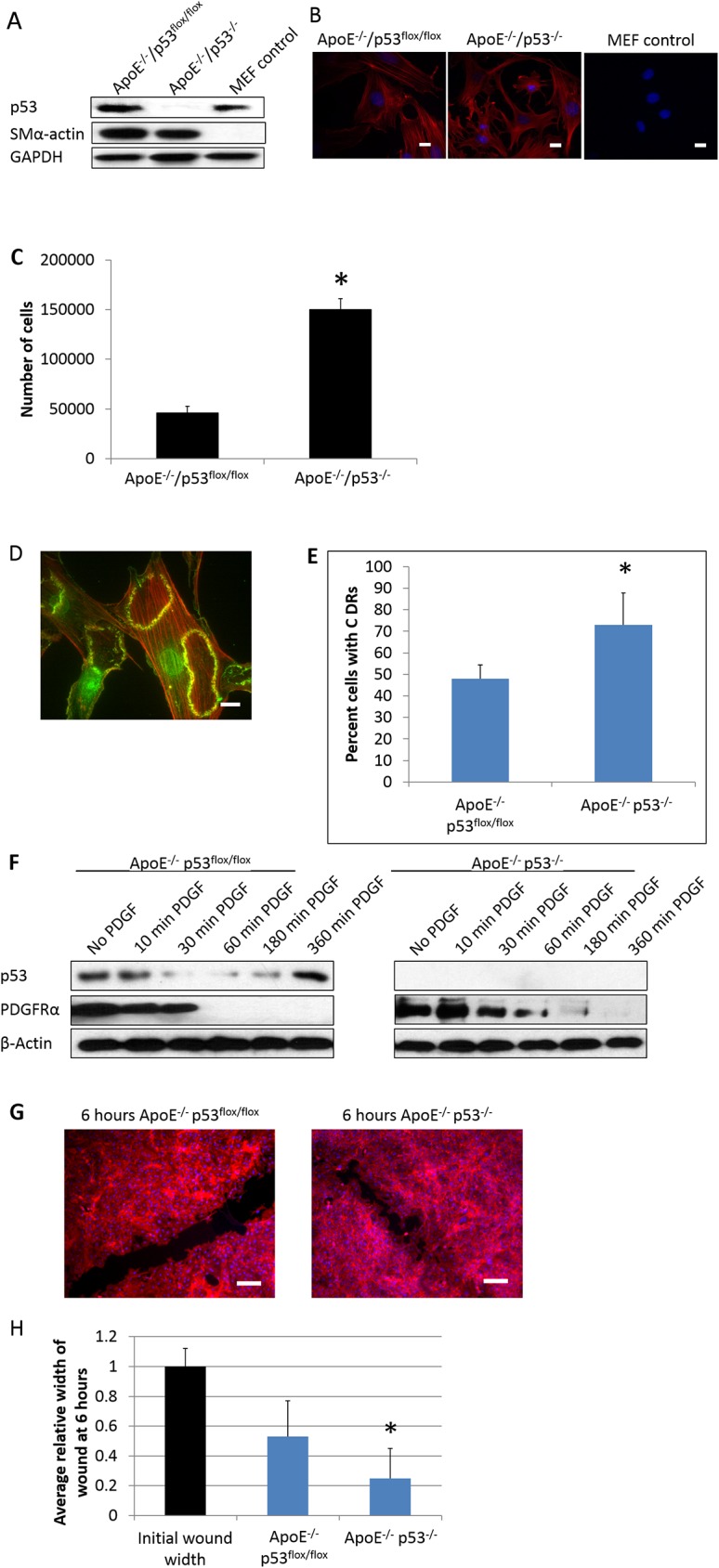
The role of p53 in PDGF-induced circular dorsal ruffle (CDR) formation and cell migration (A) Western blot of passage 2 primary VSMCs extracted from mouse aortas and Mouse Embryonic Fibroblasts (MEF), as a negative control for SM α-actin. GAPDH was used as a loading control. (B) Immune-stain of VSMCs with Cy3-SM α-actin (red) and DAPI (blue). Scale bars represent 20 μm. (C) 20,000 VSMCs cultured in a 6 well dish were counted after 2 days. Error bars represent standard deviation of 3 separate trials and * indicates p value <0.05. (D)Serum-starved VSMCs stimulated with 20 ng/ml PDGF for 10 min and stained for cortactin (green) and F-actin with TRITC-phalloidin (red) shows representative CDR formation. Scale bar represents 20 μm. (E) Serum-starved VSMCs stimulated with 20 ng/ml PDGF were time-lapse imaged for 60 min to count the percentage of cells producing CDRs. Error bars represent standard deviation from 3 separate trials. (F) Western blot of serum-starved VSMC lysates taken at various time intervals after PDGF stimulation. β-actin was used as a loading control. (G-H) VSMCs grown to 100% confluency were scratched to create a wound in the cell monolayer. 6 hours post-wound, cells were stained for F-actin with TRITC-phalloidin (red) and DAPI (blue). Initial wound width measurements were taken and compared to wound width after 6 hours and plotted as relative values. Scale bars represent 100 μm. Error bars represent standard deviation of 3 separate trials and * indicates p value <0.05.

To ensure the purity of the VSMC and the efficacy of the p53 knockout, cells from passage 2 primary VSMC were lysed and assayed by Western blot for p53 and SMα-actin, MEF (Mouse Embryonic Fibroblasts) were used as a negative control for SMα-actin (Fig 4A).

Contamination by other cells not expressing alpha-actin, such as macrophages and fibroblasts, would not have been detected by Western blot of SMα-actin alone. However, we have carried out two other assays to ascertain the purity of isolated SMC:

1. We have examined isolated SMC by immunofluorescence microscopy and have not identified cells that do not express alpha-actin as shown in Fig 4B.
2. We also showed that isolated p53-knockout SMC did not express p53 by Western blots (Figs 1C and 4A); if they were contaminated by non-SMC, p53 would have shown up in these Western blots. We are confident that even if the isolated SMC were contaminated with other cells, the level of contamination must be insignificant and will not alter the interpretations of subsequent assays.

Notably, the proliferation rate of p53-knockout VSMCs is 3 times that of the control p53^flox/flox^ cells. Briefly, 20,000 VSMCs were seeded and cultured in a 6-well dish. After 2 days in culture, the ApoE^-/-^/p53^-/-^ VSMC increased in number to 150,000 (80–90% confluent) while the ApoE^-/-^ p53^flox/flox^ sample had only 46,000 cells that took 5–6 days to reach 80–90% confluency (Fig 4C). This increased proliferation of the p53^-/-^ cells is likely due to loss of p53-mediated cell cycle regulation.

PDGFββ is the most potent chemoattractant for VSMC migration *in vitro* [4,27] and plays a crucial role in the recruitment of VSMC to the site of atherosclerotic lesions [28]. To further investigate the role that p53 plays in PDGF-induced migration of VSMC, we created primary cultures of VSMCs isolated from aortas of ApoE^-/-^ p53^flox/flox^ and ApoE^-/-^ p53^-/-^ mice.

*In vitro* wound-healing assays show that ApoE^-/-^/p53^-/-^ VSMC closed the wound 30% faster than ApoE^-/-^/p53^flox/flox^ cells (Fig 4G and 4H), indicating that p53 suppresses VSMC migration, in agreement with our previous report using either VSMC or mouse embryonic fibroblasts (MEF) [11]. Next, we investigated if p53-knockout in VSMC also affects PDGF-induced formation of circular dorsal ruffles (CDRs), which is involved in cell migration and macro-pinocytosis of surface receptors [29]. As shown in Fig 4E, both ApoE^-/-^/p53^flox/flox^ and ApoE^-/-^ p53^-/-^ primary VSMC readily form CDRs when treated with 20 ng/ml of PDGF for 10 min; however, while 70% of p53-null cells produced at least one CDR, less than 50% of p53^flox/flox^ cells were able to do so (Fig 4D and 4E).

Fig 4F shows that both the expression of p53 and PDGFR were down-regulated rapidly by PDGF-treatment. After 30 min of PDGF-treatment, only a trace of p53 is detected until after 180 min when p53 returns to baseline levels. PDGFR expression, on the other hand, disappeared after 30 min of PDGF-treatment leading to desensitization of cells to prolonged PDGF-treatment. Although it has been suggested that PDGFR is a negative transcription target of p53 and may provide the mechanism for PDGFR downregulation. However, this is not supported by results here using p53-null cells that also show similar down-regulation of PDGFR by PDGF-treatment. Although the mechanism regulating PDGFR down-regulation is not clear, it is possible that PDGF-induced CDR formation may contribute to internalization of PDGFR in agreement with reports [29] that CDR provides a rapid, global internalization of surface receptors such as integrin and growth factor receptors by macro-pinocytosis.

### p53-knockout enhances podosome formation and cell invasion in VSMC

**Fig 5:**
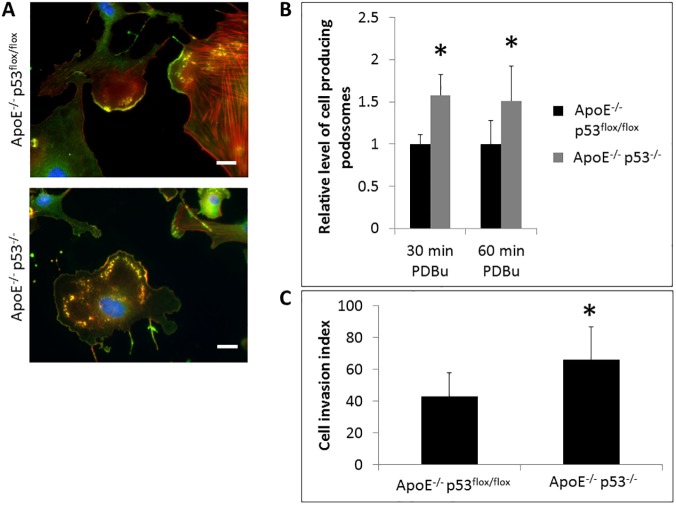
VSMC lacking p53 show increased podosome formation and cell invasion (A) Representative images of cells displaying podosomes were stimulated with 2 μM PDBu for 60 min and stained for F-actin (TRITC-phalloidin), cortactin (green), DAPI (blue). Scale bars represent 20 μm. (B) The percentage of cells producing podosomes after 30 or 60 min of PDBu exposure were plotted. (C) Cells were added to Boyden chambers with and without Matrigel using 20 ng/ml PDGF as a chemoattractant for 20 hours. Percent cell invasion = the number of cells that pass through the Matrigel / the number of cells that pass through without Matrigel. Error bars represent standard deviation from 3 separate experiments and * indicates p value <0.05.

While increased VSMC migration in p53-null VSMC may contribute to initiation of atherosclerotic plaque formation and stabilization of the fibrous cap, invasion of the extracellular matrix is required for media-to-intima migration [3,7]. As shown in Fig 3A and 3B, holes in the elastic fiber layers were observed during early lesion formation in the aortas of mice that may indicate digestion of ECM by VSMC. We investigated this further using isolated aortic VSMC cells from p53^-/-^ and p53^flox/flox^ mice. To this end, we used phorbol ester (PDBu) to induce podosome formation and ECM digestion by secretion of matrix-metalloproteases [5,30]. As shown in Fig 5A, both p53^-/-^ and p53^flox/flox^ VSMC readily formed podosomes within 30 min treatment with 2 μM PDBu. However, 50% more of the p53^-/-^ cells produce podosomes compared to the control cells (Fig 5B).

To examine whether the ability to produce podosomes also translated into increased cell invasion, we compared the invasion capabilities of the p53^-/-^ and p53^flox/flox^ VSMC using Boyden chambers and 20ng/ml PDGF as a chemoattractant. Over a 20-hour period, the p53^-/-^ VSMCs invaded through the Matrigel 50% more than the p53^flox/flox^ cells (Fig 5C). Taken together, these data strongly suggest p53 plays a role in the downregulation of cell invasion.

## Discussion

In this study, we have investigated the role of p53 in VSMC during the formation of atherosclerotic plaques in the ApoE^-/-^ mouse model fed a Western diet. By specifically knocking out p53 in VSMC, we did not detect significant differences in the overall size of the lesions between control and p53^-/-^ mice as determined by en face Oil Red O staining. In contrast, global knockout of p53 in ApoE^-/-^/p53^-/-^ mice fed a similar Western diet revealed significantly larger lesion areas than those in ApoE^-/-^/p53^+/+^ mice at 6-, 10- and 15-week time points [31]. Subsequent studies using bone marrow-derived p53 negative macrophages also showed an increase in lesion size, implicating a major involvement of macrophages [19]. These results together suggest that p53 deficiency in VSMC alone does not affect the overall size of atherosclerotic lesions; however, p53 deficiency in immune cells such as macrophages causes increase in lesion size.

We have shown, however, that there is a significant increase in the number of p53^-/-^ VSMC in the fibrous caps of atherosclerotic plaques in the early stages of plaque development. This is the first time that VSMC enrichment in fibrous caps have been shown *in vivo* in an ApoE^-/-^/p53^-/-^ mouse model. Accumulation of VSMC and collagen deposits in the fibrous caps stabilizes and protects mature atherosclerotic plaques from rupture. In contrast, high contents of macrophages, foam cells and lipids tend to destabilize them and cause their rupture, which is a major cause of acute atherosclerosis-associated myocardial infarction. Enrichment of p53^-/-^ VSMC in the fibrous cap areas suggest that down-regulation of p53 in VSMC may provide a means for plaque stabilization. This is consistent with previous *in vivo* studies. For example, adenovirus delivery of p53 induces plaque rupture in ApoE^-/-^ mice [18] and overexpression of p53 inhibits VSMC invasion in organ cultures resulting in a profound reduction in intimal thickening [16]. Conversely, viral transfer of antisense p53 oligonucleotides increases VSMC growth in rat carotid artery [14].

To determine migration potential of p53^-/-^ VSMC, we studied aortic VSMC isolated from ApoE^-/-^ /p53^-/-^ mice in culture. We demonstrated that ablation of p53 enables VSMC to migrate at a faster rate using wound healing assays and augments PDGF-induced formation of CDR, known to be involved in cell migration. These results agree with our previous *in vitro* studies using rat aortic smooth muscle cells (RASMC) showing that shRNA-knockdown of p53 or inhibition of its activity with pifithrin promotes CDR formation [4]. In contrast, up-regulation of p53 expression or activity with doxorubicin inhibits CDR formation [4]. We have also provided evidence that p53 suppresses CDR formation by upregulating PTEN resulting in inhibition of the Cdc42-N-WASP-mediated actin polymerization pathway.

In culture, aortic VSMC from ApoE^-/-^ /p53^-/-^ mice produce significantly more podosomes and are more invasive (Fig 5B and 5C), consistent with previous reports from us [3,5,6,10,11]. In those previous studies, we have identified p53 as a suppressor of Src-induced podosome formation and proposed that p53 acts by upregulating the expression of two anti-invasion regulators: caldesmon, an actin-binding protein and a negative regulator of podosome assembly [10], and PTEN that inhibits the Src-mediated PI3K-Akt and Stat3 pro-invasion pathways [11,32].

In the present studies we have focused on the roles of p53 in VSMC migration and invasion in atherosclerosis. Enrichment of VSMC in fibrous caps of atherosclerotic plaques in p53^-/-^ mice may also be caused by deregulation of VSMC proliferation and/or apoptosis. Our *in vitro* data did show that proliferation of VSMC isolated from the p53^-/-^ mice was significantly up-regulated, which is consistent with other studies [33,34]. However, Bennett et al. [35] found that abrogation of endogenous p53 activity in cultured VSMC from human coronary atherosclerotic plaque did not increase cell proliferation or support apoptosis although plaque VSMC are very sensitive to p53-mediated apoptosis, suggesting that endogenous p53 is not pro-apoptotic and over sensitivity to apoptosis of plaque VSMC is not driven by p53. These results together indeed point to the complexity of p53 regulation of proliferation and apoptosis not only in different cell types but also at various phases of atherosclerotic plaque formation and development.

In conclusion, it is generally agreed that global deficiency of p53 accelerates atherosclerosis formation in animal models that is consistent with known roles of p53 as an anti-proliferation and pro-apoptotic agent [36,37]. However, these studies do not address the role of p53 in specific cell types or in cell migration invasion in atherosclerosis *in vivo*. In this report we have shown that knockout of p53 in VSMC alone does not contribute to the overall size of atherosclerotic lesions. However, p53^-/-^ VSMC are enriched in the fibrous caps of lesions at early stages of plaque formation, which is caused in part by an increase in VSMC migration and invasion as shown by p53^-/-^ VSMC in culture having significantly higher rates of migration and producing more CDRs and invasive podosomes.

It should also be pointed out that the role of p53 in VSMC only represents one of many facets of atherosclerotic plaque formation and stability. Many questions remain to be addressed in future studies. For example, does p53 contribute to the regulation of VSMC migration in the initial stages of plaque formation, and the stability of the fibrous caps in mature plaques? Does p53 affect VSMC differentiation, e.g. changing into other cell types such as macrophage-like cells [38] and secretion of collagen in the plaque?

## References

[1] Owens GK, Kumar MS, Wamhoff BR (2004) Molecular regulation of vascular smooth muscle cell differentiation in development and disease. Physiol Rev 84: 767–801. 10.1152/physrev.00041.2003 15269336

[2] Brozovich FV, Nicholson CJ, Degen CV, Gao YZ, Aggarwal M, Morgan KG (2016) Mechanisms of Vascular Smooth Muscle Contraction and the Basis for Pharmacologic Treatment of Smooth Muscle Disorders. Pharmacol Rev 68: 476–532. 10.1124/pr.115.010652 27037223 PMC4819215

[3] Mak AS (2011) p53 regulation of podosome formation and cellular invasion in vascular smooth muscle cells. Cell Adh Migr 5: 144–149. 10.4161/cam.5.2.14375 21164280 PMC3084980

[4] Payne LJ, Eves RL, Jia L, Mak AS (2014) p53 Down regulates PDGF-induced formation of circular dorsal ruffles in rat aortic smooth muscle cells. PLoS One 9: e108257 10.1371/journal.pone.0108257 25247424 PMC4172730

[5] Mak AS (2014) p53 in cell invasion, podosomes, and invadopodia. Cell Adh Migr 8. 10.4161/cam.27841 PMC4198344 24714032

[6] Mukhopadhyay UK, Mak AS (2009) p53: is the guardian of the genome also a suppressor of cell invasion? Cell Cycle 8: 2481 10.4161/cc.8.16.9269 19657223

[7] Louis SF, Zahradka P (2010) Vascular smooth muscle cell motility: From migration to invasion. Exp Clin Cardiol 15: e75–e85. 21264073 PMC3016065

[8] Linder S, Wiesner C, Himmel M (2011) Degrading devices: invadosomes in proteolytic cell invasion. Annu Rev Cell Dev Biol 27: 185–211. 10.1146/annurev-cellbio-092910-154216 21801014

[9] Muller PA, Vousden KH (2014) Mutant p53 in cancer: new functions and therapeutic opportunities. Cancer Cell 25: 304–317. 10.1016/j.ccr.2014.01.021 24651012 PMC3970583

[10] Mukhopadhyay UK, Eves R, Jia L, Mooney P, Mak AS (2009) p53 suppresses Src-induced podosome and rosette formation and cellular invasiveness through the upregulation of caldesmon. Mol Cell Biol 29: 3088–3098. 10.1128/MCB.01816-08 19349302 PMC2682007

[11] Mukhopadhyay UK, Mooney P, Jia L, Eves R, Raptis L, Mak AS (2010) Doubles game: Src-Stat3 versus p53-PTEN in cellular migration and invasion. Mol Cell Biol 30: 4980–4995. 10.1128/MCB.00004-10 20733006 PMC2953057

[12] Guevara NV, Chen KH, Chan L (2001) Apoptosis in atherosclerosis: pathological and pharmacological implications. Pharmacol Res 44: 59–71. 10.1006/phrs.2001.0840 11516253

[13] Scheinman M, Ascher E, Levi GS, Hingorani A, Shirazian D, Seth P (1999) p53 gene transfer to the injured rat carotid artery decreases neointimal formation. J Vasc Surg 29: 360–369. 9950994 10.1016/s0741-5214(99)70389-7

[14] Matsushita H, Morishita R, Aoki M, Tomita N, Taniyama Y, Nakagami H, et al (2000) Transfection of antisense p53 tumor suppressor gene oligodeoxynucleotides into rat carotid artery results in abnormal growth of vascular smooth muscle cells. Circulation 101: 1447–1452. 10736291 10.1161/01.cir.101.12.1447

[15] Li Z, Cheng H, Lederer WJ, Froehlich J, Lakatta EG (1997) Enhanced proliferation and migration and altered cytoskeletal proteins in early passage smooth muscle cells from young and old rat aortic explants. Exp Mol Pathol 64: 1–11. S0014-4800(97)92204-8 [pii]; 10.1006/exmp.1997.2204 9203504

[16] George SJ, Angelini GD, Capogrossi MC, Baker AH (2001) Wild-type p53 gene transfer inhibits neointima formation in human saphenous vein by modulation of smooth muscle cell migration and induction of apoptosis. Gene Ther 8: 668–676. 10.1038/sj.gt.3301431 11406761

[17] Kinscherf R, Claus R, Wagner M, Gehrke C, Kamencic H, Hou D, et al (1998) Apoptosis caused by oxidized LDL is manganese superoxide dismutase and p53 dependent. FASEB J 12: 461–467. 9535218 10.1096/fasebj.12.6.461

[18] von der Thusen JH, van Vlijmen BJ, Hoeben RC, Kockx MM, Havekes LM, van Berkel TJ, Biessen EA (2002) Induction of atherosclerotic plaque rupture in apolipoprotein E-/- mice after adenovirus-mediated transfer of p53. Circulation 105: 2064–2070. 11980686 10.1161/01.cir.0000015502.97828.93

[19] van Vlijmen BJ, Gerritsen G, Franken AL, Boesten LS, Kockx MM, Gijbels MJ, et al (2001) Macrophage p53 deficiency leads to enhanced atherosclerosis in APOE*3-Leiden transgenic mice. Circ Res 88: 780–786. 11325869 10.1161/hh0801.089261

[20] Merched AJ, Williams E, Chan L (2003) Macrophage-specific p53 expression plays a crucial role in atherosclerosis development and plaque remodeling. Arterioscler Thromb Vasc Biol 23: 1608–1614. 10.1161/01.ATV.0000084825.88022.53 12842843

[21] Feil S, Valtcheva N, Feil R (2009) Inducible Cre mice. Methods Mol Biol 530: 343–363. 10.1007/978-1-59745-471-1_18 19266339

[22] Bochaton-Piallat ML, Gabbiani F, Ropraz P, Gabbiani G (1992) Cultured aortic smooth muscle cells from newborn and adult rats show distinct cytoskeletal features. Differentiation 49: 175–185. 1377654 10.1111/j.1432-0436.1992.tb00665.x

[23] Webb BA, Eves R, Crawley SW, Zhou ST, Cote GP, Mak AS (2005) PAK1 induces podosome formation in A7r5 vascular smooth muscle cells in a PAK-interacting exchange factor-dependent manner. American Journal of Physiology-Cell Physiology 289: C898–C907. 10.1152/ajpcell.00095.2005 15944209

[24] Moghadasian MH, Nguyen LB, Shefer S, McManus BM, Frohlich JJ (1999) Histologic, hematologic, and biochemical characteristics of apo E-deficient mice: effects of dietary cholesterol and phytosterols. Lab Invest 79: 355–364. 10092072

[25] Newby AC, Zaltsman AB (1999) Fibrous cap formation or destruction—the critical importance of vascular smooth muscle cell proliferation, migration and matrix formation. Cardiovasc Res 41: 345–360. 10341834

[26] Williams H, Johnson JL, Carson KG, Jackson CL (2002) Characteristics of intact and ruptured atherosclerotic plaques in brachiocephalic arteries of apolipoprotein E knockout mice. Arterioscler Thromb Vasc Biol 22: 788–792. 12006391 10.1161/01.atv.0000014587.66321.b4

[27] Grotendorst GR, Chang T, Seppa HE, Kleinman HK, Martin GR (1982) Platelet-derived growth factor is a chemoattractant for vascular smooth muscle cells. J Cell Physiol 113: 261–266. 10.1002/jcp.1041130213 6184376

[28] Boucher P, Gotthardt M (2004) LRP and PDGF signaling: a pathway to atherosclerosis. Trends Cardiovasc Med 14: 55–60. 10.1016/j.tcm.2003.12.001 15030790

[29] Orth JD, McNiven MA (2006) Get off my back! Rapid receptor internalization through circular dorsal ruffles. Cancer Research 66: 11094–11096. 10.1158/0008-5472.CAN-06-3397 17145849

[30] Burgstaller G, Gimona M (2005) Podosome-mediated matrix resorption and cell motility in vascular smooth muscle cells. American Journal of Physiology-Heart and Circulatory Physiology 288: H3001–H3005. 10.1152/ajpheart.01002.2004 15695563

[31] Guevara NV, Kim HS, Antonova EI, Chan L (1999) The absence of p53 accelerates atherosclerosis by increasing cell proliferation in vivo. Nat Med 5: 335–339. 10.1038/6585 10086392

[32] Poon JS, Eves R, Mak AS (2010) Both lipid- and protein-phosphatase activities of PTEN contribute to the p53-PTEN anti-invasion pathway. Cell Cycle 9: 4450–4454. 10.4161/cc.9.22.13936 21084866

[33] Wassmann S, Wassmann K, Jung A, Velten M, Knuefermann P, Petoumenos V, et al (2007) Induction of p53 by GKLF is essential for inhibition of proliferation of vascular smooth muscle cells. J Mol Cell Cardiol 43: 301–307. 10.1016/j.yjmcc.2007.06.001 17659301

[34] Yoo SH, Lim Y, Kim SJ, Yoo KD, Yoo HS, Hong JT, et al (2013) Sulforaphane inhibits PDGF-induced proliferation of rat aortic vascular smooth muscle cell by up-regulation of p53 leading to G1/S cell cycle arrest. Vascul Pharmacol 59: 44–51. 10.1016/j.vph.2013.06.003 23810908

[35] Bennett MR, Littlewood TD, Schwartz SM, Weissberg PL (1997) Increased sensitivity of human vascular smooth muscle cells from atherosclerotic plaques to p53-mediated apoptosis. Circ Res 81: 591–599. 9314841 10.1161/01.res.81.4.591

[36] Tabas I (2001) p53 and atherosclerosis. Circ Res 88: 747–749. 11325863 10.1161/hh0801.090536

[37] Mercer J, Bennett M (2006) The role of p53 in atherosclerosis. Cell Cycle 5: 1907–1909. 10.4161/cc.5.17.3166 16929177

[38] Gomez D, Owens GK (2012) Smooth muscle cell phenotypic switching in atherosclerosis. Cardiovascular Res. 95: 156–164. 10.1093/cvr/cvs115 PMC3388816 22406749

